# Deep Learning-Assisted Smartphone-Based Electrochemiluminescence Visual Monitoring Biosensor: A Fully Integrated Portable Platform

**DOI:** 10.3390/mi15081059

**Published:** 2024-08-22

**Authors:** Manish Bhaiyya, Prakash Rewatkar, Amit Pimpalkar, Dravyansh Jain, Sanjeet Kumar Srivastava, Jitendra Zalke, Jayu Kalambe, Suresh Balpande, Pawan Kale, Yogesh Kalantri, Madhusudan B. Kulkarni

**Affiliations:** 1 Department Electronics Engineering, Ramdeobaba University, Nagpur 440013, India; zalkej@rknec.edu (J.Z.); kalambej@rknec.edu (J.K.); 2 Department of Mechanical Engineering, Israel Institute of Technology, Technion, Haifa 3200003, Israel; rewatkarpp10@gmail.com; 3 Department of Computer Science & Engineering, Ramdeobaba University, Nagpur 440013, India; pimpalkarap@rknec.edu; 4 Computer Science & Information Systems, Birla Institute of Technology & Science Pilani, Hyderabad Campus, Hyderabad 500078, India; 5 Department of Electrical & Electronics Engineering, Birla Institute of Technology & Science Pilani, Hyderabad Campus, Hyderabad 500078, India; p20200106@hyderabad.bits-pilani.ac.in; 6 Department of Information Technology and Security, Ramdeobaba University, Nagpur 440013, India; balpandes@rknec.edu; 7 Fractal Analytics Private Limited, Pune 411045, India; 8 Citco Shared Services Private Limited, Mumbai 400072, India; 9 Department of Medical Physics, University of Wisconsin-Madison, Madison, WI 53705, USA

**Keywords:** deep learning, electrochemiluminescence, visual monitoring, biosensor, point-of-care testing

## Abstract

A novel, portable deep learning-assisted smartphone-based electrochemiluminescence (ECL) cost-effective (~10$) sensing platform was developed and used for selective detection of lactate. Low-cost, fast prototyping screen printing and wax printing methods with paper-based substrate were used to fabricate miniaturized single-pair electrode ECL platforms. The lab-made 3D-printed portable black box served as a reaction chamber. This portable platform was integrated with a smartphone and a buck-boost converter, eliminating the need for expensive CCD cameras, photomultiplier tubes, and bulky power supplies. This advancement makes this platform ideal for point-of-care testing applications. Foremost, the integration of a deep learning approach served to enhance not just the accuracy of the ECL sensors, but also to expedite the diagnostic procedure. The deep learning models were trained (3600 ECL images) and tested (900 ECL images) using ECL images obtained from experimentation. Herein, for user convenience, an Android application with a graphical user interface was developed. This app performs several tasks, which include capturing real-time images, cropping them, and predicting the concentration of required bioanalytes through deep learning. The device’s capability to work in a real environment was tested by performing lactate sensing. The fabricated ECL device shows a good liner range (from 50 µM to 2000 µM) with an acceptable limit of detection value of 5.14 µM. Finally, various rigorous analyses, including stability, reproducibility, and unknown sample analysis, were conducted to check device durability and stability. Therefore, the developed platform becomes versatile and applicable across various domains by harnessing deep learning as a cutting-edge technology and integrating it with a smartphone.

## 1. Introduction

Point-of-care testing (PoCT) technology has emerged significantly in recent years, with miniaturization, integration of advanced technologies, expanded test menus, enhanced connectivity, and disposable devices [1,2,3]. Hence, the entire testing process takes place on-site or near the patient receiving care or treatment [4,5]. It involves a variety of methodologies, including electrochemistry (EC) [6,7,8], chemiluminescence (CL) [9], electrochemiluminescence (ECL) [10,11,12,13], fluorescence (FL), and high-performance liquid chromatography (HPLC) to report the diagnostic results. However, high sensitivity, minimal background signal, ease of reaction control, simple instrumentation, and a wide dynamic range make ECL an ideal and effective tool for PoCT [14,15,16]. As a result, a wide range of PoCT–ECL-based devices have been developed, not only using diverse microfabrication technologies but also for personal or public health care and disease. Although several studies have sought to enhance the sensitivity and integration of ECL–PoCT devices, achieving complete portability, lab-free operation, reduced specialization, and full automation remains a formidable challenge. 

So far, the advancement in ECL–PoCT has mainly focused on three key domains: device fabrication, analyte detection and chemistry, and detection methodologies. In a nutshell, several fabrication technologies, including, screen printing, lithography, laser-induced graphene (LIG), and 3D printing, have been explored [17,18,19]. Likewise, it is utilized across various fields, targeting analytes ranging from disease biomarkers to environmental pollutants and food contaminants [20,21,22]. To diagnose conditions, commonly used chemistries in ECL include ruthenium-based luminophores paired with co-reactants like tripropylamine (TPA) or peroxydisulfate (S_2_O_8_^2^⁻) [23,24,25]. Additionally, in hybrid or modified ECL setups, the combination of luminol and hydrogen peroxide (H_2_O_2_) is employed for specific applications, particularly in biosensing and analytical contexts [26,27]. Finally, to detect the emitted ECL signal, techniques such as the photomultiplier tube (PMT), charge-coupled device (CCD), and electrochemical luminescence imaging (ECLI) were employed. In recent times, newly adopted technologies such as mobile phones and CMOS camera-integrated Raspberry Pi have also been utilized for ECL signal detection [28,29,30]. 

However, a lot of research work has been accomplished in all three key domains to overcome associated challenges like miniaturization, optimization of assay conditions, signal stability, background interference, accurate quantification, multiplexing, sample handling integration, and real-time monitoring with analysis [31]. Most of these challenges have been successfully addressed by adopting recent technological advancements and continued research and development efforts aim to further enhance performance. However, developing user-friendly and fast-response designs for glucometer-like devices requires addressing major technical challenges such as accessing test data with quick quantification, real-time monitoring, remote monitoring, and providing educational resources. This advancement enables individuals to manage their condition more effectively and achieve better health control [32].

**Figure 1:**
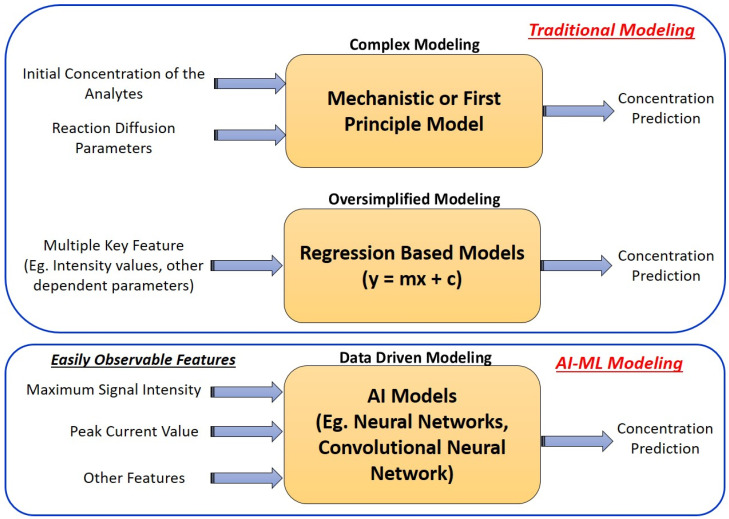
Comparative study for traditional and data-driven modeling to predict concentration.

To this end, complex mathematical modeling strategies incorporating partial differential equations (PDEs) were solved using the COMSOL^TM^ Multiphysics/MATLAB software [33]. These models optimize parameters such as system charge, reaction rate, momentum, and mass transfer. As technology advances, artificial intelligence (AI) and machine-learning (ML) techniques have been used. In ML, regression-based data-driven models were used to optimize the device parameter which improves overall accuracy. In this sense, Bhaiyya ML and his group have performed extensive studies on Laser-Induced Graphene (LIG) and 3D-printed ECL systems where they detected glucose, lactate, choline, and cholesterol [34]. The device’s testing voltage generation and the quantification of emitted ECL signals were conducted using a standalone smartphone equipped with a customized Android app interface. The various regression-based ML algorithms were used to optimize the associated parameters to improve the overall accuracy. The algorithms further provide a graphical user interface (GUI) application to calculate ECL intensity, with the cloud-based data storage facility enabling the emailing of the end results to the user. Figure 1 shows the comparative study for traditional modeling and data drive modeling to predict concentration.

Despite its unique capabilities, it still encounters challenges including data dependence, overfitting, interpretability issues, computational resource requirements, model selection complexity, bias, and fairness concerns. As an alternative to regression-based ML approaches, AI-driven algorithms would be a better option, as they improve the accuracy, sensitivity, and reproducibility of ECL assays, leading to advancements in the diagnostics process. It can extract valuable insights from complex ECL datasets, optimize experimental parameters, and facilitate real-time decision-making. It also contributes to automation, streamlining workflows, and reducing human error in ECL experiments [35,36].

Keeping in mind the advantages of AI-based ECL systems, in the present work, screen-printed single-pair electrode ECL devices were successfully fabricated and validated by performing lactate sensing. In addition, to have a wax boundary (hydrophobic and hydrophilic zones) over the paper-based substrate wax printing method was effectively used. The analytical performance of the fabricated device was explored using Luminol/H_2_O_2_-based electrochemistry with specific enzymes (lactate oxidase) to detect lactate. The important chemical reactions involved in detecting lactate are explained by the following equations [20,37]. The results demonstrate that the developed 3DP-ECL platform, when integrated with a smartphone, holds significant promise for clinical applications. It offers the potential for detecting a broad spectrum of biomarkers with high sensitivity and at a low cost.

## 2. Enzymatic Reactions

Herein, enzymatic reactions do not involve direct electron transfer at the electrode but produce H₂O₂ as a by-product. The electrochemical reactions at the electrodes include the reaction of luminol and H₂O₂ at the anode and the reduction of oxygen at the cathode. The mechanism of the enzymatic and ECL reactions can be described as follows [37].

Glucose + O_2_ → gluconololactone + H_2_O_2_ (1)

Lactate + O_2_ → pyruvic acid + H_2_O_2_ (2)

(3) $$\begin{array}{c} \mathrm{BPE}\;\mathrm{Anode}\;\mathrm{Reaction}:\\ \mathrm{Luminol}\;+\;H_{2}O_{2}\;\to \;3\text{-}\mathrm{aminophthalate}\;+\;\mathrm{hv} \end{array}$$

(4) $$\begin{array}{c} \mathrm{BPE}\;\mathrm{Cathode}\;\mathrm{Reaction}:\\ 2H_{2}O\;+\;O_{2}\;+\;4e^{-}\;\to \;4{\mathrm{OH}}^{-} \end{array}$$

## 3. Experimental Section

### 3.1. Chemicals, Materials, and Instrumentation

Luminol (97% purity), lactate (95% purity), and lactate oxidase (LOx) were procured from Sigma Aldrich, Nagpur, India. Sodium hydroxide (NaOH) was procured from Boffin Butler PVT. Ltd., Nagpur, India. Whatman filter paper (Grade 1) and conductive ink were obtained from Boffin Butler PVT. Ltd., Nagpur, India. The conductive ink used exhibits good electrical conductivity (<2.5 kΩ/25 μm at 150 °C) and has the advantage of low-temperature drying (<100 °C). Additionally, it demonstrates excellent adhesion on a wide range of substrates. A KYLIE Pro Wax100 warmer hot wax heater, VMS professional LM deluxe heavy-duty lamination Machine hot and cold A3 laminator, and REES52 buck-boost converter module type C DC-DC 5V to 3.5V/12V USB step UP/Down power supply module adjustable boost buck converter out DC 1.2V–24V were purchased from Amazon, Nagpur, India. 

Luminol is insoluble in normal water but dissolves in basic electrolytes. Thus, we prepared a luminol stock solution using the following method. Briefly, a base solution was prepared by dissolving 399 mg of NaOH in 10 mL of deionized (DI) water. Next, 80 mg of luminol was added to 47 mL of DI water, along with 3 mL of the NaOH solution, to create a 10 mM luminol stock solution. To prepare various concentrations of luminol for analysis, we diluted the stock solution using the standard dilution formula. Apart from luminol, all chemical preparations were conducted using deionized (DI) water [38].

### 3.2. Fabrication Flow for ECL Biosensor

Screen printing and wax printing methods play a key role in biosensor fabrication due to their flexibility and cost-effectiveness. Their simplicity and adaptability make them a key player in translating biosensor technology from the lab to real-world applications. Keeping the advantages in mind, herein, the ECL biosensor was successfully fabricated using these methods and tested for lactate detection. The fabrication flow is explained stepwise in the following paragraph. 

**Figure 2:**
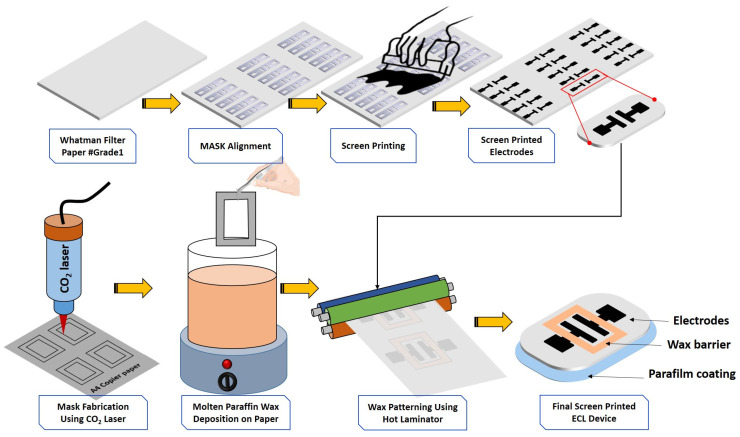
Pictorial representation of the fabricated ECL biosensor using a well-known screen and wax printing method.

First, using a laser cutter system, the mask was prepared and aligned over the paper-based substrate. Then, with conductive ink, conducting zones were prepared on the paper using screen printing. Then screen-printed devices were kept in an oven for one hour at 45 °C for curing. At the same time, to form a wax barrier (hydrophilic and hydrophobic zones), A4 paper was cut using a laser system and placed deep into the molted wax. Next, this wax-coated paper was carefully placed over the conducting zones and passed through the hot laminator. Finally, parafilm coating was performed to provide more stability to the biosensor. The complete fabrication flow is depicted as follows in Figure 2. 

### 3.3. ECL Imaging and Analysis Mechanism

**Figure 3:**
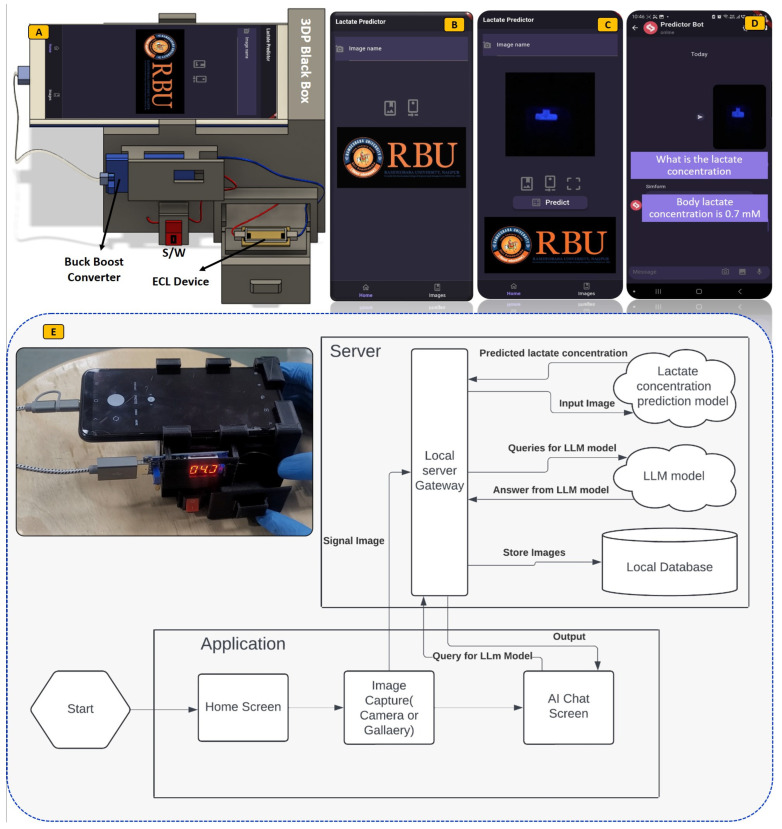
3D printed portable ECL imaging system with Android application interface. (**A**) Schematic of 3D printed portable ECL system, (**B**–**D**) android application graphical user interface for the ECL system, (**E**) Original ECL system prototype image and algorithm for developed system.

To guarantee an ECL system that is easy to use, functional in real-world settings, and manageable by non-trained personnel, a deep learning-enabled 3D-printed black box with smartphone devices has been effectively designed for the detection of bioanalytes, shown in Figure 3A. In addition, the Lactate Predictor is a user-friendly, cross-platform Flutter application designed to facilitate data acquisition and streamline the analysis process in this research. This application allows users to either capture images using their device’s camera or select images from a gallery. These images are then sent to a server via an HTTP API request, where the lactate concentration is predicted and returned to the user. Additionally, the application features an AI chatbot that assists users in analyzing lactate concentration results and addressing various queries, as shown in Figure 3B–D. A Flask server application has been developed to support this functionality. It hosts two main components: a transfer learning MobileNet model for predicting lactate concentration and the GPT4All large language model (LLM) for the chatbot. Specifically, we have utilized the “orca-mini-3b-gguf2-q4_0.gguf” model from GPT4All. 

Upon launching the app, users are presented with a home screen where they can capture or select an image and initiate the prediction process by clicking the ‘Predict’ button. When an image is sent to the server, a chat window automatically opens, enabling the user to interact with the Flask server. The server processes the image using the MobileNet model to predict lactate concentration and leverages the GPT4All model to respond to user queries. The integration of these models ensures a seamless and interactive user experience. The flowchart with a detailed explanation showing how the prediction of bioanalytes can be performed is shown in Figure 3E. The key Flutter packages used in the development of this application include image_cropper, camera, image_picker, and chatview. On the server side, the essential packages include Flask, TensorFlow, and GPT4AII. This combination of a mobile application and a robust server-side infrastructure offers a powerful tool for researchers and users to efficiently predict lactate concentrations and engage in meaningful data analysis and interpretation.

## 4. Results and Discussion

### 4.1. Parameter Optimization

Optimization of luminol, pH, applied voltage, and channel length ensures robust and sensitive ECL detection, enabling accurate analysis and quantification of analytes in various applications. The electron transfer reaction over the electrode surface is greatly influenced by the applied voltage. The rate of ECL reaction and duration of light emission can be controlled by optimizing the applied voltage. However, excessively high voltage values may lead to electrode degradation or undesirable side reactions. 

**Figure 4:**
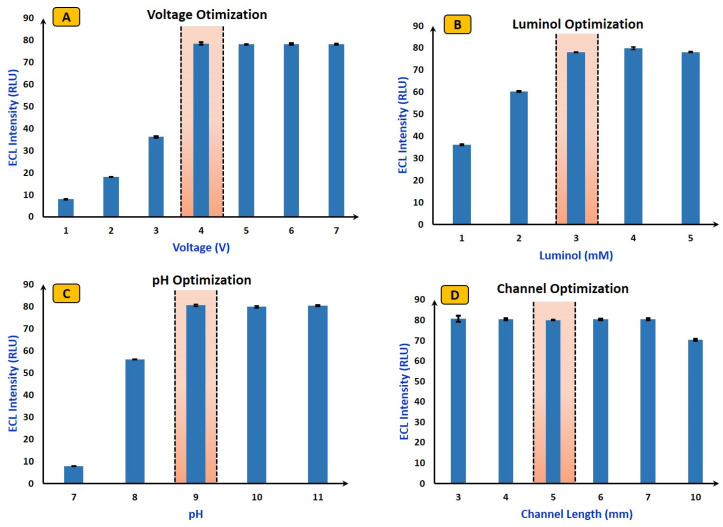
(**A**) Voltage optimization: voltage (varied from 1 V to 7 V), lactate (0.5 mM), LOx (10 Units/mL), and Luminol (5 mM). (**B**) Luminol optimization: luminol (varied from 1 mM to 5 mM), lactate (0.5 mM), LOx (10 Units/mL), and voltage (4 V). (**C**) pH optimization: pH (varied from 7 to 11 pH), lactate (0.5 mM), LOx (10 Units/mL), voltage (4 V), and luminol (3 mM). (**D**) Channel optimization: channel length (varied from 3 mm to 10 mm), lactate (0.5 mM), LOx (10 Units/mL), voltage (4 V), and luminol (3 mM). The error bar represents all experiments that were performed separately three times.

In this study, the voltage optimization was carried out by varying the potential starting from 1 V to 7 V (shown in Figure 4A), and ECL images were captured. It was noticed that, after 4 V, the ECL intensity reached saturation, with no further changes in ECL intensities. The observed results in Figure 4A can be explained by the specific conditions and design of a single-pair electrode ECL device. Although oxygen gas production occurs at 7 V or above, the ECL emission remains stable due to the efficient configuration of single pair electrode ECL device. The single-pair electrode design can easily minimize bubble interference, allowing continuous and unobstructed ECL emission. Additionally, the geometry of the device promotes rapid dissipation of bubbles away from the electrode surface, preventing significant disruption to the ECL signals. This setup ensures that the generated oxygen does not interfere with the detection process, resulting in the consistent ECL emission observed [39].

In ECL reactions, luminol plays a key role. The sensitivity of the sensors can easily improve by optimizing their concentration. A lower concentration of luminol may result in weak ECL signals, while too high a luminol concentration can lead to background noise and interference [28]. Herein, the optimization of luminol was performed by changing its concentration from 1 mM to 5 mM and the ECL signal was captured for each concentration (shown in Figure 4B). Interestingly, after reaching a concentration of 3 mM, the ECL signals became saturated, showing no further change in intensities. Hence, for further experimentation, the optimized value of luminol was used. 

Similarly, the ECL signal kinetics and the stability of intermediates deeply depend on the pH values. The optimized pH value improves the electron transfer rates which leads to enhanced ECL signal intensity [28]. The pH optimization was achieved by varying its value from 7 pH to 11 pH, and after 9 pH no change was observed in the ECL signals (shown in Figure 4C). Next, optimization of the microfluidics channel (distance between two electrodes) was performed to obtain a stable ECL signal. To improve the signal-to-noise ratio, sensitivity of detection and to get stable ECL signal channel optimization is necessary. Herein, the channel length varies from 3 mm to 10 mm, and the most stable ECL signal with less than 5% standard deviation was observed at a 5 mm channel length. Hence, a 5 mm distance was maintained through experimentation (shown in Figure 4D). Finally, incubation time optimization was carried out. The ECL signal intensity is highly dependent on the incubation time, which is the time required to produce H_2_O_2_ when lactate (1 mM) and LOx (20 Units/mL) are mixed. In our study, we varied the incubation time from 0 to 10 min in 1 min intervals and measured the ECL intensities at each interval. We observed that the ECL intensities increased linearly for up to six minutes, after which they reached saturation. Therefore, we selected an incubation time of six minutes for further experimentation.

### 4.2. Analytical Performance of ECL Biosensors

**Figure 5:**
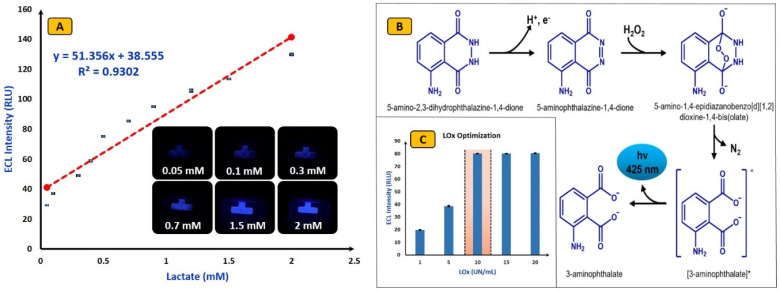
Performance analysis: (**A**) lactate detection through screen-printed ECL device. (**B**) Possible ECL chemical reactions. (**C**) LOx optimization: LOx (varied from 1 Unit/mL to 20 Units/mL), lactate (0.5 mM), LOx (10 Units/mL), voltage (4 V), and luminol (3 mM). The error bar represents all experiments that were performed separately three times. * indicates higher energy states.

Following optimization, the potential application of the fabricated ECL device was validated by conducting lactate detection. Luminol/H2O2-based electrochemistry with optimized value of lactate oxidase was used to detect lactate. Initially, the background signal was measured (no lactate), and it was observed that, in the absence of lactate, an almost negligible signal was detected. The gradual linear increment in ECL signal intensities was observed with an increment in lactate concentration. The fabricated ECL device showed a good linear range compared to the literature varying from 0.05 mM to 2 mM (from 50 µM to 2000 µM) with a limit of detection (LoD = 3.3*RSD/Slope) of 5.14 µM. The obtained results are quite interesting and show the fabricated devices with Luminol/H_2_O_2_-based electrochemistry can be used to verify applications. To obtain optimum results, the lactate LOx was optimized by varying it from 1 Unit/mL to 20 Units/mL. It was observed that, after 10 Units/mL, the ECL signal was saturated. Hence, for all experimentations, 10 Units/mL LOx concentration was used. The calibration curve, possible ECL reactions, and optimization curve for lactate oxidase are shown in Figure 5A–C, respectively.

### 4.3. Reproducibility, Stability, and Interference Study Using ECL Biosensor

**Figure 6:**
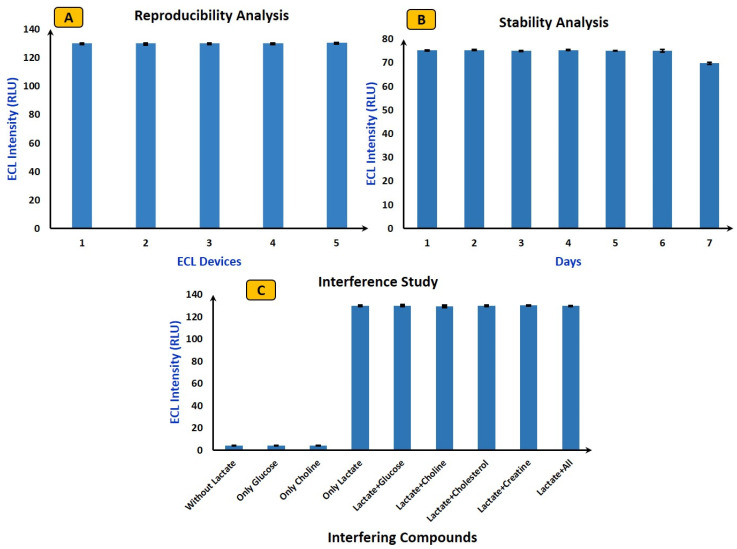
(**A**) Reproducibility analysis: lactate (2 mM), LOx (10 Units/mL), voltage (4 V), and luminol (3 mM). (**B**) Stability analysis: lactate (0.5 mM), LOx (10 Units/mL), voltage (4 V), and luminol (3 mM). (**C**) Interference study: lactate (2 mM) and glucose/choline/cholesterol/creatine (1 mM). The error bar represents all experiments that were performed separately three times.

In ECL, ensuring reproducibility, stability, and interference study of the fabricated device is crucial to obtaining reliable and consistent results. Reproducibility assesses the consistency of results across different experiments or different devices. For reproducibility analysis (shown in Figure 6A), five independent ECL devices from different batches (intra-assay) were chosen, and their luminescence responses have been examined in connection with Luminol/H_2_O_2_-based electrochemistry. The five different ECL devices from different batches exhibited relative standard deviations within an acceptable range, all falling below 5% (4.7%, 4.62%, 4.43%, 4.23%, and 4.88%). Next, in order to examine the ECL biosensors’ long-term performance and shelf life, and to guarantee their consistent functionality over time, stability studies were carried out. To carry out stability analysis, the same device was used continuously for seven days (shown in Figure 6B), with ECL signals recorded for each individual experiment. Following each use, the device was sealed and stored at 4 °C to avoid contamination. The figure reveals that, in comparison to the initial signal, over 99% of the ECL signal was consistently achieved during the first six days. However, on the seventh day, there was a notable decrease, reaching almost 90%. These results imply that, throughout the duration of a week, the ECL device performs with acceptable stability.

Finally, selectivity analysis was performed to check the anti-interference capabilities of the ECL device. It is important to carry out interference studies to identify and mitigate potential sources of interference that could affect the accuracy and specificity of ECL biosensors. Herein, the most common interferences, such as glucose, choline, cholesterol, and creatine, were selected for selectivity analysis. First, the ECL signal was measured for samples without lactate, containing only glucose and choline, and it was found that no ECL signal was observed. This is because luminol with LOx does not produce any light in the absence of lactate. Next, with optimized values of all parameters (mentioned in Section 4.2), the ECL intensity was calculated for lactate (2 mM). Subsequent separate experiments were conducted by considering every individual interfering compound, ECL images were captured, and RSD was calculated. The RSD for all interfering analytes remained below 5% (shown in Figure 6C), indicating that the device upholds excellent selectivity and remains unaffected by the introduction of interfering compounds.

## 5. Deep Learning Modeling to Validate the Analytical Performance of ECL Biosensors

### 5.1. Dataset Statistics

Following analytical performance, over 500 experiments were conducted, and deep learning-assisted ML models were trained and tested using ECL images. The dataset statistics, as shown in Table 1, reveal vital insights into its composition and distribution, which is essential for robust model training. Before training the ML model, image data augmentation techniques were implemented to enhance the dataset. Data augmentation is crucial in training deep learning models, especially when dealing with limited datasets. It involves applying various transformations to the existing data to expand and diversify the training set artificially. Data augmentation was implemented using the ImageDataGenerator from the Keras library. These techniques systematically alter images through transformations like rotation, scaling, and flipping, effectively diversifying the dataset. By augmenting the data, the model gains exposure to a broader range of scenarios and variations, thereby improving its ability to generalize to unseen data. This augmentation process enriches the dataset, mitigating overfitting issues and enhancing the model’s performance across diverse real-world conditions. Consequently, the augmented dataset reflects a more comprehensive representation of the target domain and empowers the model with increased adaptability and robustness in its predictions.

The ImageDataGenerator is configured with several augmentation hyperparameters (shown in Table 2), such as rescaling the pixel values to the range of from 0 to 1 (rescale = 1/255), shearing the images (shear_range = 0.2), zooming into or out of images (zoom_range = 0.2), and horizontally flipping images (horizontal_flip = True). These transformations help the model generalize better by exposing it to variations in the input data. The image size (img_size) is adjusted (224, 224). The batch size is also set to 32, determining the number of samples processed in each iteration during training. Runtime image data augmentation enhances model training by dynamically altering images during training, effectively increasing dataset diversity without requiring additional storage or pre-processing. This real-time augmentation improves model generalization and performance on unseen data.

### 5.2. Deep Learning Model Implementation

**Figure 7:**
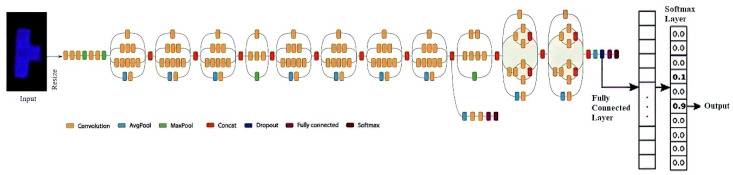
Proposed inceptionV3 architecture to predict the concentration of lactate based on ECL images.

In the InceptionV3 model architecture (shown in Figure 7), neural network neurons are structured in layers, each performing distinct transformations on its inputs. These transformations guide the flow of signals from the initial input layer to the ultimate output layer, potentially traversing multiple layers in the process. Positioned as the final hidden layer, the “bottleneck” layer encapsulates a condensed representation of information crucial for the subsequent classification task. This summarized information is a foundation for the subsequent layer responsible for actual classification. 

The efficacy of re-training the final layer for new classes in InceptionV3 stems from the realization that the information essential for distinguishing among the 1000 pre-existing classes is often transferrable and beneficial for discerning novel object categories. This adaptability underscores the model’s capacity to generalize and extend its classification capabilities beyond the original training set, showcasing the versatility and robustness of the InceptionV3 architecture in accommodating diverse classification tasks. It consists of two convolutional layers with 32 and 64 filters, respectively; each is followed by a max-pooling layer to sample the spatial dimensions down. The flattened layer reshapes the output from the convolutional layers into a one-dimensional array and then passes through two fully connected layers (Dense). The first dense layer has 128 units, with a Rectified Linear Unit (ReLU) activation function introducing non-linearity to the model. The output layer consists of a single unit, indicating a regression task, as it aims to predict a continuous numeric value. The model was compiled using the Adam optimizer with a learning rate of 0.00001, and the loss function was set to mean squared error (MSE). This configuration guides the model to minimize the average squared difference between predicted and actual values during training, enhancing its regression performance. This combination of data augmentation and a well-structured CNN facilitates practical training on a small dataset while promoting generalization to new, unseen data. The provided architecture is suitable for regression tasks that aim to predict a single continuous value.

The model was trained and tested with 80:20 dataset splitting. The dataset encompassed 10 distinct classes. During training, the model learned patterns and features within the images to make accurate predictions. The 80% training set facilitated parameter adjustments, while the 20% testing set assessed the model’s generalization to new, unseen data. This bifurcation into 10 classes enabled the model to discern and classify diverse visual elements, enhancing its ability to categorize images effectively across a range of distinct categories.

Given that all the images in the dataset were captured using a mobile camera, the InceptionV3 model is particularly well suited for this research. InceptionV3 is designed to be lightweight and efficient, making it an ideal choice for mobile and embedded devices. The research can ensure that the model performs well on the mobile camera’s dataset. Furthermore, InceptionV3 is highly effective in image classification tasks, making it a reliable choice for this research. The model runs on Google Colab, which offers access to NVIDIA T4 GPUs and features 16 GB GDDR6 VRAM and Tensor Cores for efficient deep learning tasks. T4 GPUs leverage the Turing architecture, providing substantial CUDA cores and high memory bandwidth, enhancing model training and inference speed. Users can specify GPU runtimes in their Colab notebooks, allowing seamless integration with these GPUs for accelerated machine learning workloads. 

In the image classification scenario, InceptionV3 significantly enhances traditional models like CNN, VGG16, VGG19, AlexNet, and DenseNet121. This design choice allows for a more efficient use of computational resources while maintaining high accuracy. Compared to CNN, InceptionV3 introduces lightweight depth-wise convolutions in the intermediate expansion layer, enhancing feature filtering and introducing non-linearity. This improves performance and efficiency, making it ideal for resource-constrained environments like mobile devices.

In contrast to VGG16, VGG19, and AlexNet, InceptionV3 inverted residual structure and linear bottlenecks reduce model complexity and size, enabling faster inference times without compromising accuracy. InceptionV3 surpasses these models by introducing an inverted residual structure that optimizes the use of computational resources, leading to improved performance in accuracy, model size, and computational cost. Additionally, InceptionV3’s efficient application in object detection through SSDLite and semantic segmentation with reduced DeepLabv3 models showcases its versatility and effectiveness across various tasks. InceptionV3 architecture, with its inverted residual structure and linear bottlenecks, significantly advances neural network design for image classification tasks. Its ability to outperform traditional models like CNN, VGG16, VGG19, AlexNet, and DenseNet121 while maintaining efficiency and accuracy makes it a top choice for modern image classification applications, especially with limited computational resources.

### 5.3. Comparative Analysis of Various Benchmarked Models

Transfer learning has emerged as a significant improvement in machine learning, particularly in image classification tasks. It involves using pre-trained models as a starting point for new tasks, allowing for faster training and higher accuracy. Peak accuracy for different models is compared in Table 3. 

**Figure 8:**
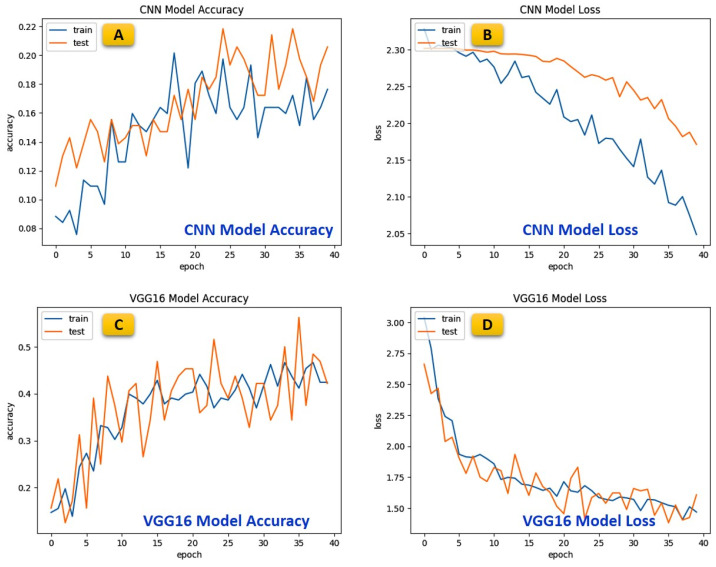

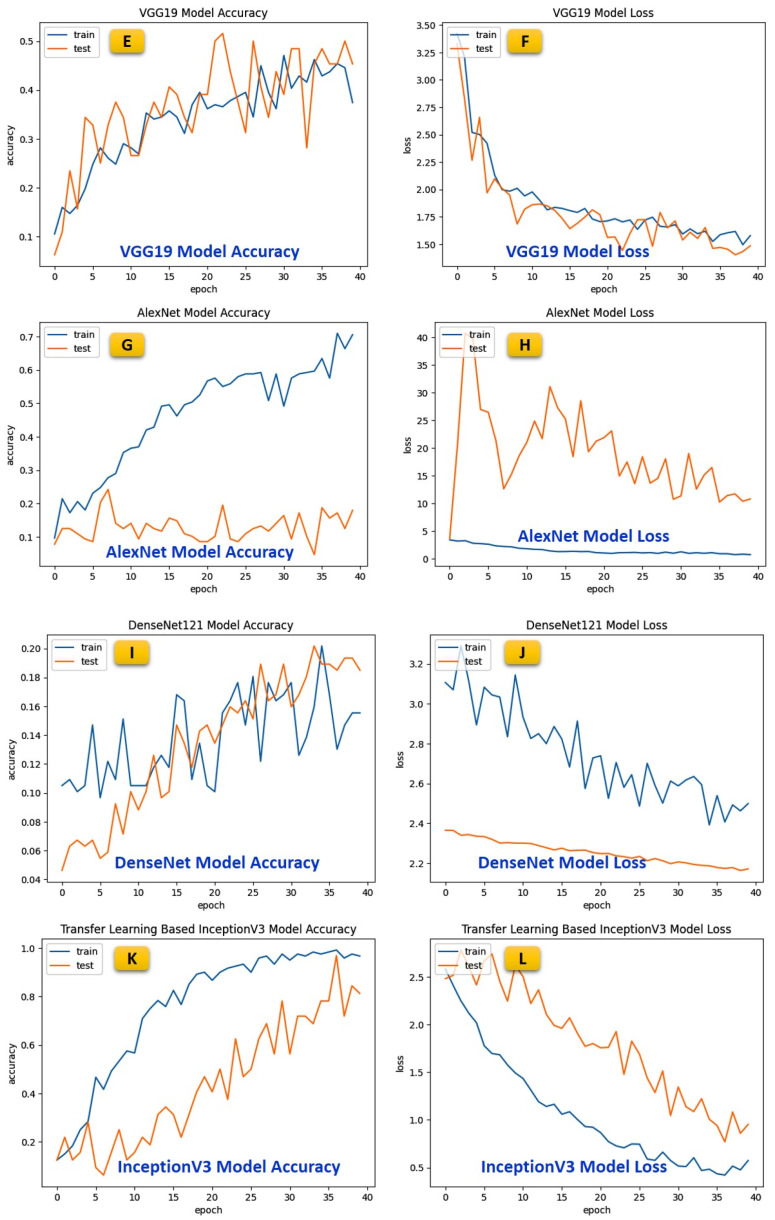
Graphical comparative analysis of various benchmarked models over accuracy and loss.

One such model that has shown remarkable results is the Transfer learning-based InceptionV3 model. The InceptionV3 model is a lightweight convolutional neural network that uses depth-wise separable convolutions to reduce the number of trainable parameters and operations, making it faster and more efficient than traditional models such as CNN, VGG16, VGG19, DenseNet121, and AlexNet, as shown in Table 3. The InceptionV3 model has a peak accuracy of 97.73%, significantly higher than the other implemented models, with 19.75, 43.70, 46.22, 20.17, and 70.59, respectively, on the same hyper-parameter. Monitoring training and validation loss is crucial for assessing the model’s performance during training. Figure 8 illustrates the training and validation loss of the implemented models. A decreasing training loss indicates effective learning, while a widening gap between training and validation losses suggests overfitting. Balancing these losses ensures the model generalizes well to new data. 

The visual representation is a crucial diagnostic tool for optimizing Transfer learning-based InceptionV3 model architecture and training parameters, ultimately contributing to improved model accuracy and reliability. The InceptionV3 model’s high accuracy is due to its ability to learn high-level features in the later layers, which are more relevant to the new data. This is achieved through fine-tuning, where the final layers of the model are re-trained with a lower learning rate to adapt to the new data. The InceptionV3 model’s architecture is also designed to perform well on mobile devices, making it an ideal choice for real-world applications. In addition to its high accuracy, the InceptionV3 model also has a lower loss than the other models. This is because the model can learn more complex features, reducing the number of misclassifications and improving the overall performance of the model. The InceptionV3 model’s success is due to its ability to learn high-level features in the later layers, making it more adaptable to new data. 

**Figure 9:**
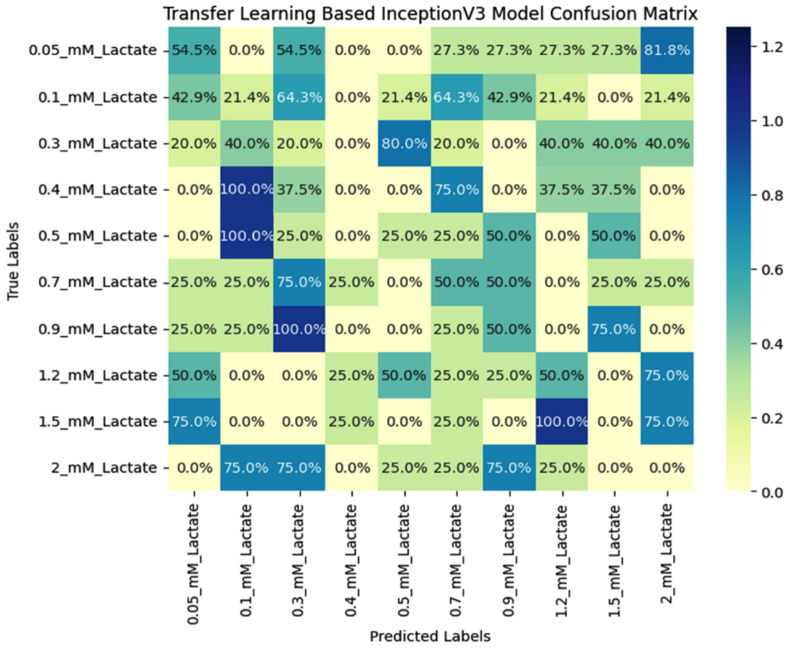
Confusion matrix for the proposed InceptionV3 model.

This is achieved through fine-tuning, where the final layers of the model are re-trained with a lower learning rate to adapt to the new data. The Transfer learning-based InceptionV3 model has shown significantly improved accuracy and loss compared to traditional models such as CNN, VGG16, VGG19, AlexNet, and DenseNet121. Its ability to learn high-level features in the later layers and its efficient architecture make it an ideal choice for real-world applications. As machine learning continues to evolve, models like the InceptionV3 play a critical role in advancing the field and improving the accuracy of image classification tasks. Additionally, the performance of the Transfer learning-based InceptionV3 model can be visualized in a confusion matrix, as shown in Figure 9, which provides a more detailed analysis of the model’s performance. The confusion matrix shows the number of true positives, false positives, and false negatives, allowing for a more comprehensive evaluation of the model’s accuracy. The high accuracy of the InceptionV3 model is reflected in the confusion matrix, with a low number of false positives and false negatives indicating that the model can correctly classify most of the data. Visualizing the model’s performance in a confusion matrix provides a clear and concise representation of the model’s accuracy and can be used to identify any areas where the model may need further improvement. The Transfer learning-based InceptionV3 model’s high accuracy, low loss, and ability to be visualized in a confusion matrix make it an ideal choice for image classification tasks.

### 5.4. Performance Evaluation of Proposed Model through Mean Absolute Error (MAE)

Mean Absolute Error (MAE) is a widely used metric for evaluating the performance of regression models, such as those predicting numerical values. These metrics provide insights into how well the model’s predictions align with the actual values in a more interpretable way than other metrics like Mean Squared Error (MSE). MAE measures the average absolute difference between the predicted and true values. The MAE is a robust evaluation measure commonly used in regression tasks, providing insight into the average absolute difference between the actual and predicted values. The formula for MAE involves calculating the absolute difference for each instance, summing these differences, and subsequently averaging them across all instances. It is calculated using the following formula:

$$MAE=\frac{1}{n}\;\sum_{i=1}^{n}\left|Y_{i}-{\hat{Y}}_{i}\right|$$

Here, n is the number of data points, $Y_{i}$ represents the actual values, and ${\hat{Y}}_{i}$ represents the predicted values. The absolute difference $\left|Y_{i}-{\hat{Y}}_{i}\right|$ is calculated for each data point, and the average is taken across all data points. 

Table 4 serves as a comprehensive snapshot of the predictive performance of the implemented model, presenting the outcomes of verification across 20 instances. This verification process involves meticulously examining the actual values corresponding to these instances and a comparison with the values predicted by the model. The detailed recording of actual and predicted values within the table provides a nuanced understanding of the model’s effectiveness and accuracy under various scenarios. Each instance within Table 2 represents a distinct set of input parameters, enabling a thorough analysis of the model’s ability to generalize and generate accurate predictions across diverse cases. This diversity in input parameters allows for a robust evaluation, assessing the model’s performance in capturing underlying patterns within the data.

The MAE is employed as a performance metric to quantitatively measure the model’s predictive accuracy. The MAE quantifies the average absolute difference between the actual and predicted values. The MAE is calculated for each of the 20 instances, reflecting the precision of the model’s predictions for individual cases. The subsequent step involves computing the average MAE across all instances, providing an aggregate measure of the model’s overall predictive accuracy. The reported average MAE for the implemented model is 2.975525. This numerical value carries significant implications for the proposed model. As a quantitative indicator, the average MAE reflects the model’s ability to predict outcomes with high precision. A lower MAE signifies that the predicted values closely align with the actual values, indicating the model’s efficacy in capturing and reproducing the underlying patterns present in the data. Including MAE as a metric in model compilation is a crucial step in the training process. During model training, the objective is to minimize the MAE by adjusting the model’s parameters. This optimization ensures that the predicted values closely match the actual values in the training dataset. Monitoring the MAE during training is essential for assessing the model’s convergence and making informed decisions about its effectiveness. 

## 6. Unknown Sample Analysis and Its Validation Using ML

Finally, we tested the practical use of the device and its ability to predict lactate concentration using deep learning by analyzing unknown samples. First, we prepared five different lactate concentrations within the linear range using commercially available lactate. These samples were tested in the lab to obtain their concentration values. Then, we used our fabricated ECL device to test the same samples and took ECL images for each concentration. We calculated the concentration of the unknown lactate samples using a calibration curve, which matched well with the lab results. Next, we fed the ECL images into a deep-learning model (InceptionV3) to predict lactate concentration. The predictions closely matched both the actual lab results and those from the ECL device. These results indicate that the deep learning-assisted ECL device is capable of real-time applications. The analysis of unknown samples using this system is shown in Table 5. 

Following Table 6 shows the in-depth comparative analysis of AI-assisted ECL systems for different applications. 

## 7. Conclusions

In summary, a cost-effective smartphone-based electrochemiluminescence (ECL) platform with integration of deep learning was developed and successfully used for ECL imaging. The conducting zones were fabricated using screen printing, while hydrophilic and hydrophobic zones were created over paper-based substrate using a wax printing methodology. As a template, lactate detection was carried out using a portable ECL platform and the results were encouraging (linear range = from 50 µM to 2000 µM, LoD = 5.14 µM), highlighting the potential applications of the platform across diverse fields. In addition, with the development of an intuitive Android application, we ensure accessibility and ease of use for practitioners. Overall, this work is a major advancement toward the democratization of biosensing technology, providing a flexible approach with broad applications in environmental monitoring, healthcare, and other fields.

## Figures and Tables

**Table 1:** Dataset statistics before and after data augmentation.

Class	Before Data Augmentation			After Data Augmentation		
	Test	Train	Total	Test	Train	Total
0.1_mM_Lactate	9	36	45	90	360	450
0.3_mM_Lactate	9	36	45	90	360	450
0.4_mM_Lactate	9	36	45	90	360	450
0.05_mM_Lactate	9	36	45	90	360	450
0.5_mM_Lactate	9	36	45	90	360	450
0.7_mM_Lactate	9	36	45	90	360	450
0.9_mM_Lactate	9	36	45	90	360	450
1.2_mM_Lactate	9	36	45	90	360	450
1.5_mM_Lactate	9	36	45	90	360	450
2.0_mM_Lactate	9	36	45	90	360	450
**Total**	**90**	**360**	**450**	**900**	**3600**	**4500**

**Table 2:** Hyperparameters for the proposed model.

Parameter	Value
input shape	224 × 224
optimizer	SGD, Adam
learning rate	0.001
pooling	GlobalAveragePooling2D
activation function	ReLU
dropout	0.5
padding	ZeroPadding2D
epochs	40
verbose	1, 2
validation steps	8
include top	False
layer trainable	False
output layers	Dense, 10
shuffle	True
loss	categorical_crossentropy
metrics	Accuracy

**Table 3:** Comparative analysis of various benchmarked models.

Epoch/Model	CNN	VGG16	VGG19	DenseNet121	AlexNet	InceptionV3
**10**	12.61	30.25	28.99	10.50	35.29	53.33
**15**	15.55	39.92	34.45	11.76	49.16	75.83
**20**	12.18	39.92	39.50	10.50	52.52	90.00
**25**	19.75	39.08	38.66	14.71	57.98	93.33
**30**	14.29	36.97	36.13	16.81	58.82	97.50
**35**	17.23	43.70	46.22	20.17	59.66	97.50
**40**	17.65	42.44	37.39	15.55	70.59	97.73
**Peak Accuracy**	19.75	43.70	46.22	20.17	70.59	**97.73**

**Table 4:** Predictive performance of the implemented model.

Test Instance	Actual Values	Predicted Value	Mean Absolute Error Value
1	104.857	102.89	1.967
2	106.65	110.14	3.49
3	107.499	110.36	2.861
4	105.799	101.97	3.829
5	110.573	105.23	5.343
6	109.688	106.55	3.138
7	108.303	104.31	3.993
8	106.65	110.14	3.49
9	110.137	107.4	2.737
10	105.22	105.58	0.36
11	36.552	37.246	0.694
12	37.882	40.606	2.724
13	40.497	40.953	0.456
14	38.685	38.787	0.102
15	39.5	41.141	1.641
16	124	128.482	4.482
17	125.05	128.713	3.663
18	126.53	119.174	7.356
19	129.8	127.125	2.675
20	122.9325	127.442	4.5095
**Average Mean Absolute Error Value**			**2.975525**

**Table 5:** Unknown sample analysis and prediction through ML.

Analyte	Actual Value (mM)	LAB Testing (mM)	Testing Using ECL Device	Corresponding ECL Image	ML Prediction
**Lactate**	0.2	0.22	0.24	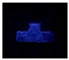	0.2
	0.35	0.34	0.42	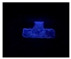	0.4
	1.1	1.18	1.2	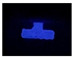	1.2
	1.35	1.42	1.4	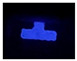	1.3
	1.8	1.9	1.85	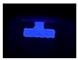	1.8

**Table 6:** Comparative analysis of AI-assisted ECL systems for different applications.

Sr. No.	Device Fabrication Method	Chemistry Used	LoD	AI-ML Models Used	Application	Real Sample Analysis	Ref. No
1.	Screen-printed electrodes	Ru(bpy)_2_^+3^/TPrA	-	Random forest and Feedforward neural network	Ru(bpy)_2_^+3^	-	[10]
2.	3D Printing	Luminol/H_2_O_2_	0.04 mM for glucose and 0.1 mM for lactate	Regression-based ML models	Glucose, Lactate	Blood serum	[13]
3.	3D Printing	Luminol/H_2_O_2_	0.49, 0.01, 0.09, and 0.3 mM	Regression-based analyses were carried out for the prediction	cholesterol, choline, lactate, and glucose	Blood serum	[17]
4.	ITO glass	Luminol/H_2_O_2_	14 mM for glucose, 40 mM for lactate, and 97 mM for choline	-	Glucose, lactate, choline	Blood serum	[20]
5.	Conventional three-electrode system	Molecularly imprinted polymer	0.25 µM	Deep Learning	Furosemide	Human urine	[35]
6.	3D Printing	Luminol/H_2_O_2_	0.5 mM	-	Lactate	Sweat	[40]
7.	Screen-printed electrodes	Ru(bpy)_2_^+3^/TPrA	-	Single or Multilayer Neural Net	phenolic compounds	-	[41]
8.	Screen-printed electrodes	Luminol/H_2_O_2_	5.14 µM	Deep Learning	Lactate	Commercial Lactate	This work

## Data Availability

The original contributions presented in the study are included in the article, further inquiries can be directed to the corresponding authors.
